# Medical and Physical Intelligence

**Published:** 1820-11

**Authors:** 


### Medical and Physical Intelligence.

ANATOMICAL *discovery respecting the Lymphatic Vessels.*—Dr. VINCENZ FOHMANN, Prosector at the Anatomical Theatre at Heidelberg, announces, in the Salzburgh *Medizinisch-Chirurgische Zeitung*, (No. 46, June 1820,) a discovery respecting the lymphatic vessels, that seems calculated to elucidate some important points in physiology.* In his researches, during last winter, respecting the intimate structure of the intestines of man and inferior animals, he found, by accident, in the large intestine of a lion which he dissected, a net-work of lymphatic vessels which had not been remarked in any animal, and which, on being injected with mercury, appeared to him *to inosculate directly with veins:* (ihm unmittelbar in *venen uberzugehen sehien.*) This discovery led him to make new researches in other animals, and he found similar lymphatic vessels in other *carnivores;* namely, the *procyon lotor* and the common *dog:* but here he could not clearly ascertain the passage of the mercury into the veins. He repeated, in the month of April, the injection of the same lymphatics in the *phoca vitulina*, and what he could not effect by means of the *vasis chyliferis*† *of the large intestine*, he effected perfectly by those of the small intestine; then, after having filled all the *lymphatics of this intestine*, and having pursued them to the *pancreas aselli*, he found some small *vasa efferentia*, by which the quicksilver had passed into the mesenteric vein. He repeated the injection in a second *phoca*, and found the metal pass into the same vein as in the foregoing experiment. He on this occasion put a ligature on all the mesenteric vessels, at some distance beyond the large mesenteric glands; and he found that those glands, as well as the veins, were filled with the mercury. He has witnessed the same facts in the dog, cow, and horse; but has not yet had an opportunity of examining the state of those parts in man: but he considers that other physiologists will investigate this subject; and he makes a question whether the thoracic duct really receives all the chyliferous vessels, or whether the names of Pecquet and Mascagni have caused a supposition to he admitted as a fact.

Dr. Fohmann announces that he intends to publish, almost immediately, a work on the true disposition and development of the intestinal glands of men and inferior animals, in which the facts above stated will be more particularly developed.

---

* It is a remarkable circumstance, that Professor HARLESS, of the University of Bonn, had formed a notion that such an anatomical structure probably existed, before this discovery of Dr. Fohmann, as he states in his work (which we shall notice particularly in the next half-yearly Review of the Progress of Medicine), *Ueber und Gegen den Neueren Empirismus in der Physiologie und Medicin*, seit 59; such a structure, he thinks, being calculated to explain and reconcile the apparently opposing results of the experiments of several eminent physiologists respecting absorption.

† This is Dr. Fohmann's own expression; but, on speaking of the vessels in question in the lion, he uses the term *lymphgefassen:* he, indeed, employs the terms *chyliferous* and *lymphatic* vessels synonymously.

*Vegetable Antidotes to Poison.*—Dr. Chisholm, in a paper read to the Society at Geneva, states that the juice of the sugar-cane is the best antidote known for arsenic. It has been tried upon various animals in the West Indies with complete success, and always succeeds. Its power in the island of Nevis is generally known.

Dr. Chisholm also mentions the singular powers of a plant, well known to the Indians, as a remedy for the ophthalmia: it is called *akouscrounie* and *warannie* by them, and *eye-root* by the white people. It grows in La Guyane, in the neighbourhood of Demerara, in a sandy soil, and is a species of bignonia, which Dr. C. has since called ophthalmica. An Indian prepares the remedy from the roots of the plants, by first stripping off the brown epidermis, and then separating a fibrous pulpy part immediately beneath: this he presses on cotton so as to collect the juice, and then, by means of a paper funnel, conveys a drop or two of it into the eye. This is repeated once a-day for three or four days, in which time the cure is generally completed. Dr. Chisholm had occasion, in his own practice, to apply this remedy in three cases; and, having only the dry root, he rasped off the outside, and then made a strong infusion of the part beneath. Six drops of this infusion were introduced into each eye once a-day, and in six days' treatment they were perfectly cured, though they had suffered for many weeks previously.

---

*On the Poison of the Viper.*—M. Configliacchi has been engaged on experiments with this poison. It was obtained by pressing the vesicles between the canine teeth into a watch-glass, and applied by means of a needle.

He established, in the most positive manner, that this poison had no effect, except when introduced into the blood-vessels; flour-pills were dipped into the poison, and swallowed by birds, without producing any injury to them.

One object was to ascertain the effect of voltaic electricity on birds poisoned by this venom. Some birds dead, but still warm, were subjected, with others killed either by breaking the neck, suffocation, or decapitation, to the powers of a pile of twenty-four pair of plates, excited by a solution of sulphate of alumine, one pole being connected with the spinal marrow, and the other with a muscle of the knee. The result was, that the irritability of the muscles was considerably diminished in those animals killed by the poison, its duration not being more than one-fourth of that of the other animals, or even one-sixth of that of the decapitated birds. It was also so weak, that four times the quantity of plates did not produce an equal effect in them.

Another result was, that when poisoned birds, not yet dead, were submitted to voltaic action, their death was hastened. The mean of three experiments gave six minutes as the difference between the death of poisoned birds electrified and not electrified.

It was also ascertained that birds poisoned by prussic acid, more or less strong, as laurel-water concentrated to various degrees, gave the

1

same results, except that the duration either of the pain or of the irritability of the muscles was much shorter than with the viper poison.—*Journal of Science, &c.*

---

A case in which the *Cæsarean Operation* was performed with a favourable issue to the mother, is related in the *Annali Universali di Medicina* of Dr. Omodei, (April 1820,) by Dr. Gensana of Saluzzo. The subject was a labouring peasant, thirty-two years of age, and had been five days in labour of a mature child, (it is not stated whether or not it was her first,) when the advice of a surgeon was required.

"The fetus, according to the account of this surgeon, Mr. Alliprandi, was wedged in between the *ossa ilia*, and the mouth of the uterus was nearly closed, from being, as well as the vagina, extremely tumefied and inflamed, in consequence of the repeated and improper manual efforts of an inexpert country midwife.

"The surgeon immediately employed blood-letting, tepid fomentations, and emollient clysters. No good arose from these means; and, the patient becoming affected with convulsions, the surgeon soon determined to resort to the Cæsarean operation, although he could not obtain the advice of any other surgeon, nor the aid of any assistant.

"He made an incision along the side of the linea alba through the integuments, parted aside the muscles (*dilatati i muscoli*), and, opening into the uterus, extracted a fetus nearly in a state of putrefaction. He remarked, that the uterus was inflamed in an *extraordinary degree*, as well as the *whole of the abdominal viscera.*

"After this operation the lochiæ appeared, to which, in the progress of time, was joined some purulent matter. The wound cicatrized perfectly in a short space of time; and a tumor which presented in the left groin, which was opportunely opened, gave vent to pus collected in it, and was reduced and firmly cicatrized in twenty days. The patient in the mean time was re-established, became again pregnant, and had a most fortunate parturition."*

---

The application of a ligature to the common carotid artery, for aneurism of its external branch, has lately been performed with success, (for the second time, in Germany: it had been before practised by Professor Walther, late of Landshut, now of Bonn,) by Dr. Holscher, surgeon to the court at Hanover. The patient was a printer, twenty-three years of age. The tumor appeared suddenly, without any evident cause, and rapidly increased in size. The patient had recovered from the operation, which had been followed by no extraordinary symptoms, in about ten weeks: "he has resumed his ordinary work, and no longer experiences the vertigo, nor the pains in his head, which troubled him before the operation."

---

* We have translated the above account very closely, (almost verbally,) much more so than is compatible with the more graceful idioms of the English language: but, as the statement is so very vague and unsatisfactory, we have chosen not to alter the turn of a single expression.

An account of what seemed to be the remains of a fetus found in the body of a girl after death, dispersed in tumors situated about the mesentery, was read at a late sitting of the Medical Society of Paris, by M. Olivry. The case seemed to be of the same kind as that of the boy whose case was related by Mr. Highmore; of the girl described by Dr. Philips, of Andover; and of the boy Bisneau, whose body is preserved in the cabinet of the Ecole de Medecine at Paris. The girl died at the age of seventeen, after having suffered for about six months from pains in her belly. "She fell a victim," says the reporter, "to a caries of the vertebral column, with steatomatous engorgements in the mesentery; as dissection of the body, made four hours after death, in presence of my *confrere* M. Goueffes, who had seen the patient with me, showed." By accident, one of the steatomatous tumors was carried home by M. Olivry; when, on examining it, he found it to contain a bone, apparently the petrous portion of a temporal, and a tooth. He could not resort to another examination of the body, to ascertain whether or not the other tumors contained other structures serving to make up the remainder of a fetus.

---

M. Pagenstecher (in Tromsdorff's Journal) says, that a concentrated infusion of violets may be preserved good for a long time, if it be exposed, in a corked bottle, to the action of boiling water for a quarter of an hour; it is then to be taken from the water, and set aside without having been uncorked. This process was first proposed by M. Appert.

---

*Oxide of Chlorine.*—M. Dobereiner has completed the series of the combinations of muriatic acid with oxygen, by the discovery of the oxide of chlorine, which was wanting in that series. He obtains it by means of oxy-muriate of potash and muriatic acid, the action of which is favoured by a gentle heat. It might be possible to obtain by the same process, and by a due proportion of those substances and a proper temperature, the different compounds of chlorine and oxygen, except the second super-oxygenated acid discovered by Stadion.

---

Professor Brera states in his Journal, (*Nuovi Commentaria di Medicina e di Chirurgia*, No. 2, 1820,) that Dr. Salvatori writes to him from Petersburgh, to say that he has employed the leaves and flowers of the *campanula graminifolia* in epilepsy, with the most fortunate results, in many cases. The following is the formula in which he has ordinarily employed it:

℞. *Herb. et flor. campanul. graminifol. unc. semis: infunde per horam in aquæ fervidæ lib. una; deinde coletur; et sumat vasculum ter de die, scilicet mane, ante prandium, et vespere ante somnum.*

### REPORT OF DISEASES.

RHEUMATISM, which was stated in our last Report to be of unusually frequent occurrence, has become still more prevalent during the last month; and it has assumed an extremely acute form in many instances. One remarkable instance of metastasis of disease of this kind, has recently occurred to our observation. Pains were first felt in the joints of the upper and lower extremities and in the loins; these disappeared after two days, and were succeeded by very intense pain apparently in the stomach, which continued about twelve hours, and then symptoms of inflammation of the synovial membrane of one of the knee-joints came on, and has given rise to a collection of fluid in its cavity. The remedy, in the generality of cases, found to be the most effectual amongst those which have been employed at one of the public Dispensaries, is a combination of calomel and opium administered freely. The ordinary prescription for adults is two scruples of calomel and eight grains of opium, made into eight pills, two of which are taken night and morning, or three times a-day if the disease be very severe. One man, labouring under very acute rheumatism, deviated from his instructions, and took eight such pills in sixteen hours: the pains were completely removed, and no bad effect produced. After the acute stage has passed away, colchicum becomes singularly beneficial; and, in obstinate cases, it frequently answers well to give from fifty to one hundred drops of the vinum colchici at night, and cinchona during the day. We spoke in favour of this combination of remedies in a former Report; and, though the two substances may be supposed, on hypothetical reasoning, to have opposite medical qualities, continued experience has made us satisfied that the combination of the two will in many cases remove the disease, when either, alone, is unsuccessful, and when many other medicines have been ineffectual.

The same causes which have produced so much rheumatism, will likewise account for the increasing prevalence of pulmonary affections; though by far the greater proportion of patients having diseases of this kind have previously had some chronic disorder of the lungs: indeed, in an extensive list of them, there has been but one case of acute pneumonia in an individual who had previously been in apparent health.

The following case is sufficiently rare to merit particular notice. A female, about twenty-three years of age, a domestic servant, who had enjoyed good health and not suffered any remarkable moral distress, became affected with what appeared like well-marked phrenitis, though not of an intense form. She was treated in the first instance by an intelligent apothecary in the ordinary manner: bleeding, general and local, was employed to a *moderate* extent; the fever and intolerance of light disappeared about the ninth day, but delirium still remained, and has continued to the present time, which is a month and a few days from the attack of the disease. There are no violent paroxysms of fury, nor were there any in the acute stage of the disease; but the perceptions are almost constantly and generally erroneous, and there is a considerable degree of wildness of look and manner. The mother of this woman became affected with puerperal mania after her first parturition, at the age of twenty-six, and died at the end of the second month of the disease.

Amongst children, by far the most prevalent disease has been measles, generally mild, but occasionally followed by diarrhœa or cough, and sometimes by both. Scarlatina has likewise appeared, but it does not seem disposed to spread extensively.

About a dozen cases of fever of a typhoid character has occurred amongst the children brought to a public medical institution. In general, the disease has been attended from the beginning with some obvious local determination, and some cough has almost always been present. When the head has been affected, advantage has ensued from the application of leeches behind the ears; but the lungs have not been so readily and easily relieved. In a few cases, ulcerations, disposed to become gangrenous, have occurred about the lips and alæ of the nose in those patients; and in one child a spot on the chin, about a third of an inch in diameter, sloughed, and separated by ulceration. The only deaths that have occurred in this class of patients, were of children affected in this manner.

---

### MONTHLY CATALOGUE OF MEDICAL BOOKS.

### METEOROLOGICAL JOURNAL.

By Messrs. William Harris and Co. 50, Holborn, London.

*From August 20 to September 19, inclusive.*

| Day of Month. | Moon. | Rain gauge | THERM. | | | BAROM. | | DeLuc's Hygro. | [illegible] | WIND. | | ATMOSPHERIC VARIATION. | | |
|---|---|---|---|---|---|---|---|---|---|---|---|---|---|---|
| Aug. | | | | | | | | | | | | | | |
| 20 | | ‘21 | 55 | 64 | 52 | 29·67 | 29·88 | 54 | 45 | NW | E | Fine | | |
| 21 | | ‘34 | 54 | 57 | 49 | 29·83 | 29 79 | 57 | 55 | ENE | NNE | Rain | | |
| 22 | | ‘19 | 54 | 56 | 48 | 29·82 | 29·92 | 57 | 57 | NE va. | NNE | Rain | Cloudy | Fine |
| 23 | | | 55 | 60 | 53 | 30·10 | 29·17 | 54 | 55 | NE | NE | Fine | | |
| 24 | ○ | | 56 | 63 | 52 | 30·14 | 30·08 | 55 | 53 | SW | SW | Fine | | |
| 25 | | ‘12 | 60 | 64 | 53 | 29·88 | 29·72 | 57 | 62 | SW | WSW | Cloudy | | Rain |
| 26 | | ‘03 | 62 | 66 | 50 | 29·67 | 29·66 | 59 | 58 | WSW | SW | Fine | Rain | Cloudy |
| 27 | | ‘08 | 56 | 64 | 53 | 29·74 | 29·65 | 55 | 55 | NW va. | WSW | Fine | | Rain |
| 28 | | ‘02 | 58 | 62 | 50 | 29·53 | 29·65 | 54 | 53 | NW va. | WSW | Fine | Show’ry | Fine |
| 29 | | | 57 | 64 | 50 | 29·70 | 29·81 | 55 | 53 | WNW | WNW | Fine | | Cloudy |
| 30 | ☽ | | 56 | 60 | 50 | 29·93 | 30·01 | 53 | 54 | W | NW | Fine | Cloudy | Fine |
| 31 | | | 56 | 60 | 48 | 30·06 | 30·11 | 54 | 54 | ENE | NE | Fine | Cloudy | Fine |
| Sep. | | | | | | | | | | | | | | |
| 1 | | | 55 | 61 | 49 | 30·08 | 30·06 | 52 | 55 | NE | NE | Fine | | |
| 2 | | | 56 | 62 | 53 | 30·06 | 30·09 | 54 | 53 | NNE | NNE | Fine | | |
| 3 | | | 58 | 63 | 49 | 30·14 | 30·19 | 55 | 53 | NNE | N | Fine | | |
| 4 | | | 53 | 60 | 49 | 30·16 | 30·15 | 54 | 53 | NE | ESE | Fine | | |
| 5 | | | 54 | 61 | 49 | 30·16 | 30·07 | 54 | 53 | SSE | ESE | Fine | | |
| 6 | | | 55 | 63 | 48 | 30·07 | 30·11 | 52 | 53 | SE | E | Fine | | |
| 7 | ● | | 57 | 64 | 53 | 30·11 | 30·16 | 53 | 54 | E | SW | Fog | Fine | |
| 8 | | | 60 | 66 | 50 | 30·23 | 30·35 | 53 | 52 | NNW | E | Cloudy | Fine | |
| 9 | | | 57 | 68 | 51 | 30·36 | 30·32 | 53 | 52 | SSW | WSW | Fine | | |
| 10 | | | 59 | 66 | 53 | 30·30 | 30·28 | 54 | 54 | W | WSW | Fine | | |
| 11 | | | 64 | 72 | 57 | 30·26 | 30·24 | 56 | 55 | ESE | ESE | Fine | | |
| 12 | | | 66 | 75 | 54 | 30·23 | 30·21 | 56 | 5[illegible] | SE | SE | Cloudy | Fine | |
| 13 | | | 63 | 70 | 57 | 30·18 | 30·11 | 56 | 54 | ESE | ESE | Fine | | |
| 14 | | | 65 | 73 | 58 | 29·91 | 29·8[illegible] | 57 | 56 | S | WSW | Fine | | Cloudy |
| 15 | ☾ | ‘17 | 64 | 67 | 51 | 29.71 | 29·94 | 57 | 52 | WSW | W | Fine | Show’ry | Fine |
| 16 | | | 57 | 66 | 53 | 29·99 | 30·03 | 54 | 54 | W | W | Fine | | Cloudy |
| 17 | | ‘65 | 59 | 65 | 50 | 29·97 | 29·86 | 53 | 55 | WSW | WSW | Cloudy | | Rain |
| 18 | | ‘81 | 53 | 61 | 40 | 29·57 | 29·78 | 55 | 53 | NW va. | WNW | Rain | Fine | |
| 19 | | | 46 | 57 | 45 | 29·95 | 30·05 | 52 | 52 | NW | WNW | Fine | | |

The quantity of rain fallen in August, is 1 inch and 36-100ths.

### METEOROLOGICAL JOURNAL.

By Messrs. William Harris and Co. 50, Holborn, London.

*From September* 20 *to October* 19, *inclusive.*

| Day of Month. | Moon. | Rain guage | THERM. | | | BAROM. | | DeLuc's Hygro. | | WIND. | | ATMOSPHERIC VARIATION | | |
|---|---|---|---|---|---|---|---|---|---|---|---|---|---|---|
| Sep. | | | | | | | | | | | | | | |
| 20 | | ‘36 | 51 | 57 | 43 | 29·92 | 29·54 | 52 | 55 | SW | W | Fine | Rain | |
| 21 | | ‘11 | 50 | 60 | 48 | 29·45 | 29·51 | 52 | 53 | W | NW | Fine | Rain | Fine |
| 22 | ○ | | 56 | 64 | 53 | 30·00 | 30·01 | 55 | 56 | NW | W | Fine | | |
| 23 | | | 60 | 65 | 57 | 30·00 | 29·96 | 60 | 61 | WSW | SW | Fine | | |
| 24 | | ‘10 | 59 | 65 | 50 | 29·75 | 29·81 | 62 | 55 | WSW | WSW | Rain | Show'ry | Fine |
| 25 | | | 51 | 60 | 43 | 29·69 | 29·76 | 54 | 53 | W | NW | Fine | | |
| 26 | | | 48 | 55 | 38 | 29·89 | 30·16 | 53 | 55 | N | NW | Fine | | |
| 27 | | ‘08 | 44 | 55 | 50 | 30·12 | 30·10 | 54 | 53 | W | WNW | Cloudy | Rain | Fine |
| 28 | | | 54 | 59 | 53 | 30 09 | 30·06 | 55 | 60 | WSW | SW | Fine | Cloudy | Show'ry |
| 29 | ☽ | ‘03 | 54 | 62 | 44 | 30·00 | 30·22 | 61 | 59 | NNE | NE | Rain | Fine | |
| 30 | | ‘01 | 50 | 59 | 46 | 30·12 | 30·03 | 60 | 62 | E | SSW | Fog | Show'ry | Fine |
| Nov | | | | | | | | | | | | | | |
| 1 | | | 50 | 60 | 48 | 30 19 | 30·32 | 59 | 57 | WSW | W | Fine | | |
| 2 | | | 51 | 59 | 47 | 30·41 | 30·59 | 56 | 54 | NNW | WNW | Fine | | |
| 3 | | | 51 | 60 | 42 | 30·53 | 30·57 | 56 | 55 | NNE | NE | Fine | | |
| 4 | | ‘02 | 45 | 56 | 49 | 30·53 | 30·42 | 54 | 59 | NNE | N E | Fine | | Show'ry |
| 5 | | | 55 | 59 | 49 | 30·33 | 30·30 | 58 | 60 | ENE | N E | Fine | | |
| 6 | | | 53 | 59 | 47 | 30·27 | 30·22 | 58 | 59 | NE | NNE | Fine | | |
| 7 | | | 51 | 57 | 47 | 30·23 | 30·25 | 58 | 60 | ENE | NE | Fine | | |
| 8 | ● | | 50 | 55 | 55 | 30·26 | 30·23 | 59 | 59 | E | NE | Fine | | |
| 9 | | | 49 | 53 | 44 | 30·23 | 30 19 | 57 | 55 | ENE | NE | Cloudy | | |
| 10 | | | 47 | 53 | 45 | 30·15 | 30·09 | 54 | 55 | NE | NE | Cloudy | | |
| 11 | | | 47 | 51 | 43 | 30·07 | 30·02 | 54 | 55 | NE | NE | Cloudy | | |
| 12 | | | 45 | 50 | 38 | 30·08 | 30·04 | 54 | 53 | N | NNW | Fine | | |
| 13 | | | 43 | 48 | 39 | 30·02 | 29·95 | 53 | 56 | NW | SSE | Fog | Fine | |
| 14 | | ‘05 | 43 | 47 | 45 | 29·71 | 29·46 | 56 | 60 | S | SSE | Cloudy | | Show'ry |
| 15 | ☾ | ‘20 | 51 | 59 | 44 | 29·07 | 29·20 | 54 | 61 | S | WSW | Rain | | Fine |
| 16 | | ‘25 | 46 | 49 | 40 | 29·21 | 29·19 | 62 | 56 | WSW | SW | Rain | | Stormy |
| 17 | | ‘09 | 43 | 48 | 39 | 29·11 | 29·01 | 57 | 59 | SW | SW | Fine | Cloudy | Fine |
| 18 | | ‘03 | 43 | 49 | 39 | 29·19 | 29·26 | 61 | 60 | WSW | WNW | Fine | Show'ry | |
| 19 | | ‘09 | 43 | 48 | 38 | 29·15 | 29·05 | 60 | 60 | WSW | SW | Fine | | Rain |

The quantity of rain fallen in September, is 2 inches and 42·100ths.

